# Determination of free and conjugated bile acids in serum of Apoe(−/−) mice fed different lingonberry fractions by UHPLC-MS

**DOI:** 10.1038/s41598-019-40272-8

**Published:** 2019-03-07

**Authors:** Tannaz Ghaffarzadegan, Sofia Essén, Phebe Verbrugghe, Nittaya Marungruang, Frida Fåk Hållenius, Margareta Nyman, Margareta Sandahl

**Affiliations:** 1 Food for Health Science Centre, Kemicentrum, Lund University, PO Box 124, SE-221 00 Lund, Sweden; 2 Centre for Analysis and Synthesis, Department of Chemistry, Kemicentrum, Lund University, PO Box 124, SE-221 00 Lund, Sweden; 3 Present Address: Food Technology, Engineering and Nutrition, Kemicentrum, Lund University, PO Box 124, SE-221 00 Lund, Sweden

## Abstract

Bile acids (BAs) are known to be involved in cholesterol metabolism but interactions between the diet, BA profiles, gut microbiota and lipid metabolism have not been extensively explored. In the present study, primary and secondary BAs including their glycine and taurine-conjugated forms were quantified in serum of Apoe−/− mice by protein precipitation followed by reversed phase ultra-high-performance liquid chromatography and QTOF mass spectrometry. The mice were fed different lingonberry fractions (whole, insoluble and soluble) in a high-fat setting or cellulose in a high and low-fat setting. Serum concentrations of BAs in mice fed cellulose were higher with the high-fat diet compared to the low-fat diet (20–70%). Among the lingonberry diets, the diet containing whole lingonberries had the highest concentration of chenodeoxycholic acid (CDCA), ursodeoxycholic acid (UDCA), tauro-ursodeoxycholic acid (T-UDCA), α and ω-muricholic acids (MCA) and tauro-α-MCA (T-α-MCA), and the lowest concentration of tauro-cholic acid (T-CA), deoxycholic acid (DCA) and tauro-deoxycholic acid (T-DCA). The glycine-conjugated BAs were very similar with all diets. CDCA, UDCA and α-MCA correlated positively with *Bifidobacterium* and *Prevotella*, and T-UDCA, T-α-MCA and ω-MCA with *Bacteroides* and *Parabacteroides*.

## Introduction

Bile acids (BAs) are synthesized from cholesterol in the liver and stored in the gallbladder to be secreted in the duodenal lumen upon food intake in order to facilitate fat digestion. When the BAs reach the colon, the microbiota transform primary BAs to secondary BAs. This transformation is highly dependent on the microbiota composition, which in turn is due to the dietary intake, e.g. content of fat and type of dietary fiber. Part of the secondary BAs is reabsorbed into the liver, conjugated and then transported into the circulation. This means that changes in the colonic BA profile caused by diet may be reflected in blood.

**Figure 1:**
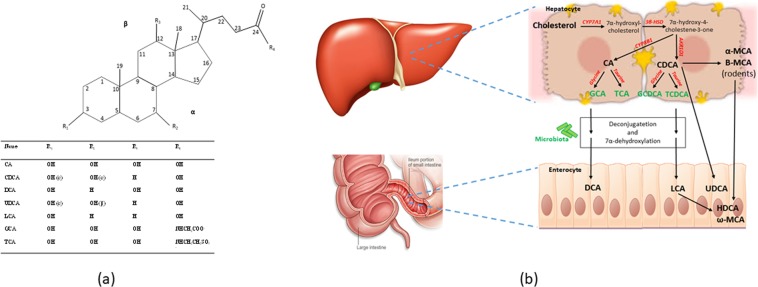
(**a**) Chemical structure of different BAs (**b**) BAs synthesis pathway.

The primary BAs in humans are cholic acid (CA) and chenodeoxycholic acid (CDCA), both produced in the hepatocytes through the oxidation of cholesterol^1^ (Fig. 1). The primary BAs in rodents, α and β-muricholic acid (MCA), and the secondary BA, ω-MCA, are responsible for elimination of cholesterol from the body^2–4^. Primary BAs are conjugated in the liver with the amino acid glycine or taurine through the terminal carboxylic group, which means that the molecules will become negatively charged at physiological pH with enhanced water solubility. Most BAs in mice are taurine-conjugated (95%), while the most abundant conjugated BAs in humans are glycine-conjugated^5^. Secondary BAs – deoxycholic acid (DCA) and lithocholic acid (LCA) – are formed through microbial 7α-dehydroxylation of CA and CDCA, respectively^6^. Ursodeoxycholic acid (UDCA), which is another secondary BA, is also converted from CDCA, but through microbial oxidation and epimerization^7^. Germ-free mice display markedly elevated levels of MCA compared to conventionally raised mice^8^. Furthermore, the gut microbiota regulate BA metabolism by reducing the levels of a farnesoid X receptor (FXR) antagonist, tauro-β-MCA^8^. The diet can modify the gut microbiota composition, which implicates that effects of dietary factors on BA profiles need to be characterized further.

Up to 90% of the primary BAs in human adults are produced through the classical neutral pathway in the liver, while the remaining 10% are synthesized via the acidic pathway^9–11^. In the classical pathway, cholesterol is hydroxylated by the enzyme cholesterol 7α-hydroxylase (CYP7A1), forming 7α-hydroxycholesterol^12^, which in turn goes through several reactions before forming CA and CDCA (Fig. 1). Synthesis of CA is catalyzed by sterol 12α-hydroxylase (CYP8B1)^9^, while a cytosolic enzyme called 3-oxo-∆4- steroid 5β-reductase (AKR1D1) accelerates the reaction leading to the synthesis of CDCA^13^.

In humans, the BA pool size is kept comparatively stable at about 3–5 g through the enterohepatic circulation^14^. In this process about 95% of the BAs that have entered the small intestine are reabsorbed from the terminal ileum back to the liver via the portal vein. The remaining BAs enter the large intestine where they undergo extensive bacterial transformation before absorption/excretion^15,16^.

It is increasingly suggested that some BAs are involved in carbohydrate and lipid metabolism and these have been suggested to improve hyperglycemia^17^, insulin resistance^18^, intestinal inflammation^19^, cholestasis disease^20^ and gut barrier permeability, whereas others stimulate tumor development^21^ and colon cancer^22^. It is therefore valuable to observe profiles and amounts of serum BAs to be able to assess diseased conditions and diagnoses. The complex physico-chemical attributes of the BAs, e.g. polarity and lipophilicity, may also play important pharmaceutical roles. Hence, there is a growing need for methods to identify and quantify BAs in biological matrices.

Previously we developed a method that included both extraction and analysis of free BAs in cecum material of rats, a method that can also be applied to feces of humans^23^. With this method, it was shown that various types of dietary fiber, as well as the same fiber but with different physico-chemical properties, produced different cecal BA profiles in rats^24^. Furthermore, the amount of β-glucan degraded by the microbiota in the cecum of rats was of great importance for the primary and secondary BAs formed^25^. It is therefore interesting to see whether similar differences in the raw material composition are also reflected in the BAs of blood. If so, the method would be applicable for the study of different types of alterations with human dietary interventions. As changes in the BAs profile can be used as biomarkers of disease, it is important to have a comprehensive analytical method for accurate determination of BA composition in blood.

In recent decades, several analytical methods have been developed for quantitative analysis of BAs in different biological samples^26–28^. Separating, identifying and quantifying all BAs is however challenging, due to their complex chemical structures and relatively low concentration (µmol/L) in complex biological matrices^29,30^. Gas chromatography combined with mass spectrometry (GC-MS) has been used extensively for analysis of BAs^31,32^. GC analysis, however, is tedious as it requires monotonous pre-analytical procedures including extraction, purification and hydrolysis of conjugated BAs and derivatization^33,34^. Another conventional technique for analyzing BAs is high-performance liquid chromatography (HPLC) coupled either to UV^35,36^ or fluorescence detectors^37^. An advantage of HPLC-UV compared to GC is that derivatization is not required, but the poor selectivity of UV detector makes this method unreliable^38^. In recent years, liquid chromatography-mass spectrometry (LC-MS) has become more popular for determination of free and conjugated BAs in different biological samples such as plasma, liver, bile and feces of humans and rodents^26,39–42^. Mass spectrometric detection is a good alternative both for identification and quantification of BAs, as this technique provides better selectivity and a lower detection limit as compared to conventional LC-UV^41,43^.

The objective of the present work was to study whether ultra-high-performance liquid chromatography–mass spectrometry (UHPLC-MS) could be used for the determination of free and conjugated BAs in serum of Apoe−/− mice given three lingonberry fractions and to relate the BA composition to the microbiota composition. Intake of lingonberries has previously been shown to reduce blood cholesterol levels and change hepatic BA gene expression, cecal short-chain fatty acid (SCFA) profile, and gut microbiota composition in Apoe−/− mice compared to the same mice given a control diet containing cellulose^44^. Apoe−/− mice are genetically modified to obtain high cholesterol levels and atherosclerotic plaques with age. Previous studies have shown that dietary factors can affect atherogenesis in these mice by microbial modulation of BAs synthesis in the liver^44,45^. Both lingonberries and resveratrol increase the numbers of *Lactobacillus*, *Bifidobacterium* and *Akkermansia* in the gut, of which the first two bacteria possess enzymes for bile-salt hydrolase.

To clarify whether effects of lingonberries on atherosclerosis were mediated by an altered BA profile, we investigated BAs in Apoe−/− mice. Additionally, to be able to connect properties of the raw material to the BA composition, whole lingonberries were separated into water-soluble and insoluble fractions^46^, which differed in composition and functional properties. Soluble fiber, for example, is known to affect the microbiota composition, through its capacity to be fermented to a greater extent than insoluble dietary fiber^26^. With the UHPLC-MS method used, 21 BAs most commonly found in the serum were separated and selectively detected during a 16-minute gradient. Furthermore, with this method, a CSH column was used; this is a C18-column with cationic character and, to our knowledge, had not been used before. Such a column provides a second retention mechanism in addition to standard C18 column.

## Results

**Figure 2:**
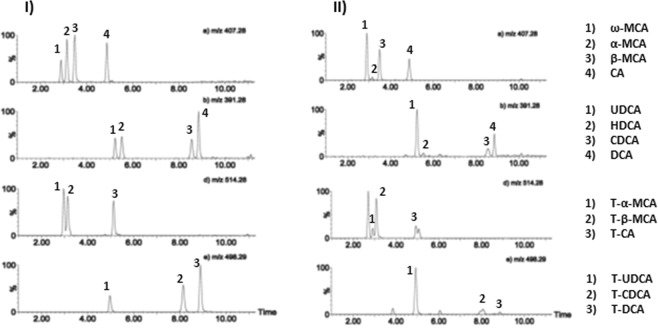
Ion chromatograms of free and taurine-conjugated BAs. The intensity of each chromatogram is normalized to 100%. I) standard 10 ng/mL and II) serum sample from Apoe*−/−* mice fed with whole lingonberries.

Ion chromatograms of the BAs in a standard solution as well as in a serum sample of Apoe−/− mice are depicted in Fig. 2. The quantitative results of the serum BA profiling are presented in Table 1.

**Table 1:** Free and conjugated BAs in the serum of Apoe*−/−* mice (ng/mL) fed whole lingonberries and their separated soluble and insoluble fractions and the control diets.

Bile acids	Control – Low Fat	Control – High Fat	Whole Lingonberry	Soluble Lingonberry^a^	Insoluble Lingonberry
**Unconjugated**					
CA	99.6 ± 15.0	90.7 ± 12.0	71.5 ± 7.0	70.0 ± 4.8	114.0 ± 16.2
α-MCA	13.3 ± 0.8	17.2 ± 0.8	23.8 ± 3.0^†††^	13.0 ± 0.8	18.3 ± 1.5
β-MCA	40.3 ± 3.5	92.7 ± 12.8^†††^	78.7 ± 6.1^††^	54.6 ± 6.7	81.6 ± 10.8^††^
ω-MCA	62.6 ± 4.9	100.6 ± 14.2	157.0 ± 25.3*††††	59.7 ± 2.8	64 ± 7.0
CDCA	27.3 ± 0.1	29.1 ± 0.4	34.5 ± 1.5*^††††^	28.0 ± 0.2	32.5 ± 1.3^††^
DCA	80.4 ± 6.0	116.7 ± 7.5	62.1 ± 16.0*	112 ± 6.0	110.4 ± 13.0
HDCA	20.9 ± 0.6	20.4 ± 0.6	26.3 ± 3.4	19.7 ± 0.3	19.5 ± 0.6
UDCA	22.9 ± 0.7	34.6 ± 3.5	62.7 ± 11.2*††††	24.3 ± 1.5	35.0 ± 2.3
LCA	<RL	<RL	<RL	<RL	<RL
**Glycine-conjugated**					
G-CA	15.9 ± 0.1	16.2 ± 0.1	16.5 ± 0.2	15.9 ± 0.1	16.3 ± 0.2
G-LCA	25.2 ± 0.01	25.2 ± 0.01	25.2 ± 0.02	25.2 ± 0.01	25.2 ± 0.02
G-CDCA	19.2 ± 0.2	19.0 ± 0.5	19.5 ± 0.2	19.2 ± 0.6	19.7 ± 0.5
G-DCA	18.0 ± 0.01	18.2 ± 0.07	18.1 ± 0.05	18.1 ± 0.06	18.1 ± 0.05
G-UDCA	13.5 ± 0.02	13.5 ± 0.03	13.6 ± 0.01	13.6 ± 0.01	13.6 ± 0.01
**Taurine-conjugated**					
T-CA	132.2 ± 15.2	175.6 ± 16.7	99.8 ± 10.5*	252.1 ± 31.8^††^	151.0 ± 28.9
T-α-MCA	57.3 ± 6.3	207.9 ± 24.8^††††^	182.6 ± 20.7^††††^	163.6 ± 18.0^††^	98.9 ± 8.1**
T-β-MCA	30.3 ± 1.6	55.2 ± 1.8	66.0 ± 7.5^†††^	48.2 ± 2.6	68.6 ± 10.8^†††^
T-UDCA	43.1 ± 1.6	87.1 ± 5.7^††††^	76.3 ± 3.1^††††^	53.1 ± 2.4****	46.6 ± 3.3****
T-CDCA	29.0 ± 0.5	32.7 ± 1.1	30.5 ± 0.9	35.9 ± 3.3^††^	31.0 ± 1.2
T-DCA	36.5 ± 1.9	38.8 ± 2.1	28.6 ± 1.0**^†^	36.4 ± 0.4	37.4 ± 2.3
T-LCA	<RL	<RL	<RL	<RL	<RL

Results are given as means ± SEM, n = 7.

Mean values were significantly different from the High Fat control group: *P < 0·05, **P < 0·01, ***P < 0·001, ****P < 0·0001 (one-way ANOVA and Dunnett’s test).

Mean values were significantly different from the Low Fat control group: ^†^P < 0·05, ^††^P < 0·01, ^†††^P < 0·001,^††††^P < 0·0001 (one-way ANOVA and Dunnett’s test).

^a^6 mice completed the study, 3 of them were outliers.

### Concentration of unconjugated BAs

The concentration of most unconjugated BAs was higher (P < 0.001 for β-MCA) in the serum of mice fed the HF control diet as compared to the LF control diet (Table 1). When including whole lingonberries in a HF diet, the serum concentration of some free BAs increased further and was significant (P < 0.01–P < 0.0001) for the MCAs, CDCA and UDCA. The increase was to a certain extent also reflected in the group fed the insoluble lingonberry fraction, which was significant for β-MCA and CDCA (P < 0.01). No significant effects were seen in the group fed the soluble lingonberry fraction.

By comparing results in groups fed lingonberry diets with results from the HF control group, the concentrations of serum ω-MCA, CDCA and UDCA were also higher (P < 0.05) in mice fed whole lingonberries, while the serum concentration of DCA was lower (p < 0.05). There were no effects of the soluble and insoluble lingonberry fractions when compared to the HF control diet (Table 1).

### Concentration of conjugated BAs

The concentration of glycine-conjugated BAs in serum was very similar between groups, while taurine-conjugated BAs were affected to a greater extent (Table 1). Thus, the group fed the HF control diet generally had higher serum concentrations of taurine-conjugated BAs than the group fed the LF control diet, which was significant for T-α-MCA and T-UDCA (P < 0.0001). A similar increase in serum concentrations was seen in the group fed the whole lingonberries compared to the LF group, except for T-CA and T-DCA, which instead decreased (P < 0.05 for T-DCA). These trends were reflected to a certain extent also in groups fed the soluble and insoluble lingonberry fractions, although with less significance. Thus, the soluble lingonberry fraction had higher concentrations of T-CA, T-α-MCA and T-CDCA (P < 0.01). The group fed the insoluble lingonberry fraction significantly increased T-β-MCA (P < 0.001) (Table 1).

Compared to the group fed the HF control diet, the serum concentration of taurine-conjugated BAs generally decreased in groups fed lingonberries (Table 1). Thus, in the group fed whole lingonberries it was significant for T-CA (P < 0.05) and T-DCA (P < 0.01) and, for groups fed the soluble and insoluble fractions, T-UDCA was significant (P < 0.0001). Furthermore, for groups fed the insoluble fraction, T-α-MCA reached significance (P < 0.01).

### Comparison of different lingonberry diets

**Figure 3:**
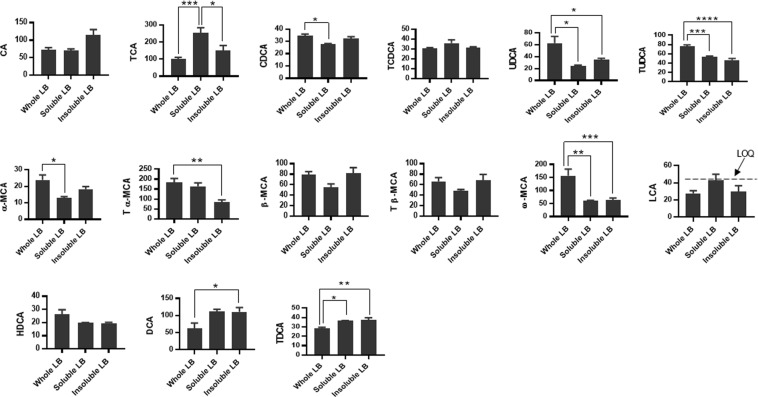
Concentration of serum BAs in Apoe*−/−* mice (ng/mL) fed whole lingonberries, soluble and insoluble fractions of the same lingonberries. Mean values were significantly different between different lingonberry groups: *P < 0.05, **P < 0.01, ***P < 0.001, ****P < 0.0001 (one-way ANOVA and Tukey’s test).

The whole lingonberry diet seemed to have a higher capacity to affect the serum BAs’ composition in Apoe−/− mice compared to diets containing soluble and insoluble lingonberry fractions (Fig. 3). Thus, of the free and taurine-conjugated BAs the serum concentration of CDCA, UDCA, T-UDCA, α-MCA, T-α-MCA and ω-MCA was highest in the group fed whole lingonberries, while the concentration of DCA and T-DCA was lower. The concentration of T-CA followed a different pattern and the whole lingonberry group had the lowest amount of this BA compared to the other lingonberry diets, while the diet containing the soluble lingonberry fraction exhibited the highest concentration of T-CA.

The serum concentrations of CA, T-CDCA, β-MCA, T-β-MCA and HDCA were similar with all diets containing lingonberries.

### Gut microbial taxa associated with BAs and experimental diets

**Figure 4:**
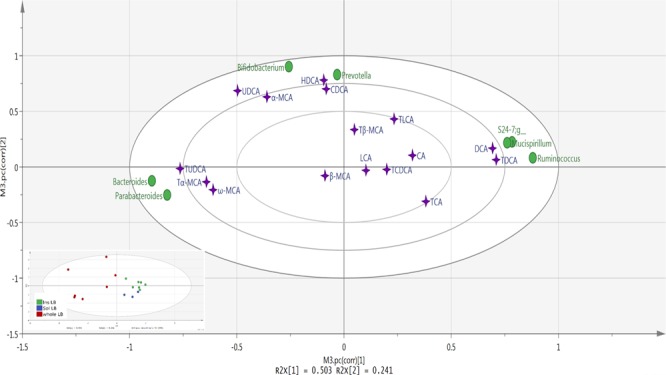
PLS loading and score scatter plots of BAs and gut microbiota. Plots illustrate correlations between BAs (purple stars) and the gut microbiota (green circles) in Apoe*−/−* mice fed diets containing whole lingonberries and soluble and insoluble fractions of the same lingonberries. A larger distance from the origin (0.0) shows a stronger correlation. The score scatter plot to the bottom left indicates how each mouse is placed with respect to the PLS loading and according to the groups (green = insoluble lingonberry; blue = soluble lingonberry; red = whole lingonberry).

**Figure 5:**
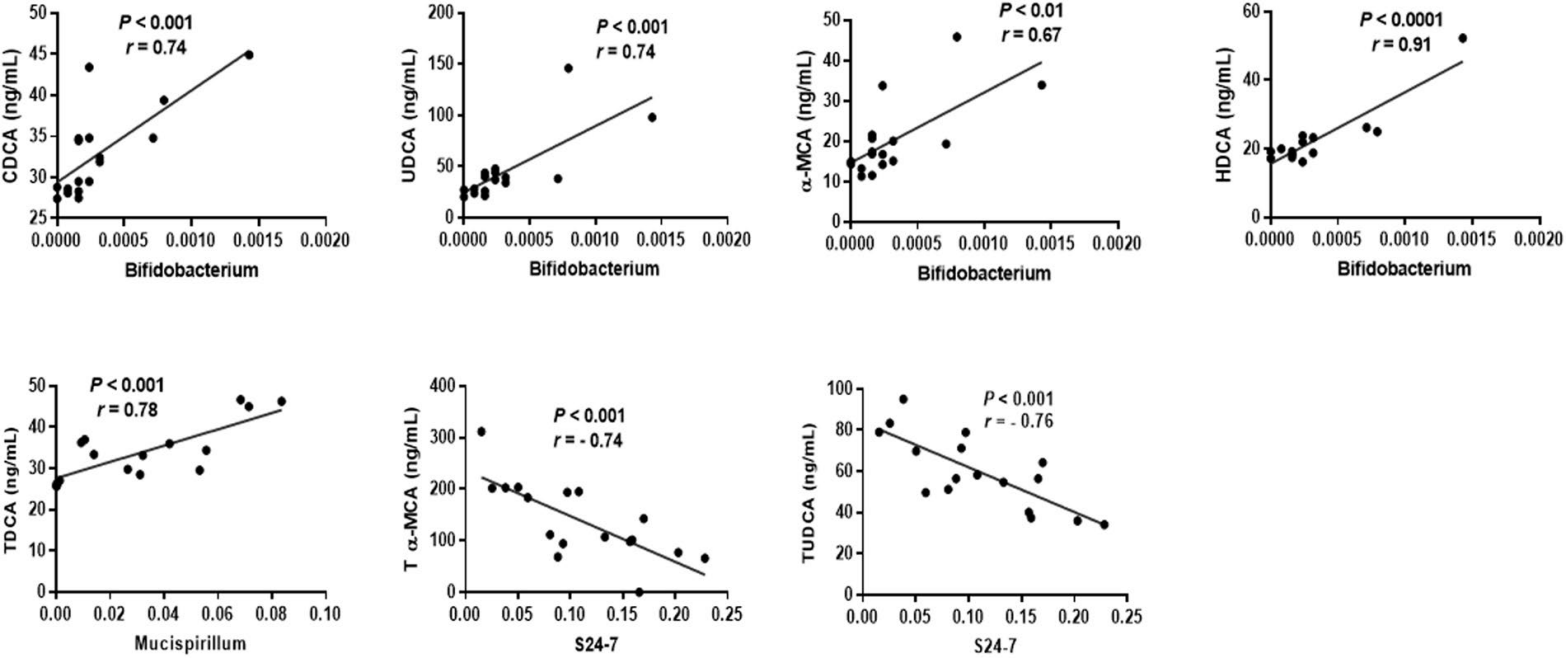
Strong correlation between BAs and selected microbiota in Apoe*−/−* mice (r-values > 0.7). X-axis represents the relative abundance of each microbiota.

To investigate how different serum BAs were related to gut microbiota, a partial least squares (PLS) analysis was performed for mice consuming the different lingonberry diets. PLS loading and score scatter plots (Fig. 4) showed that CDCA, UDCA, α-MCA and HDCA formed one cluster together with *Bifidobacterium* and *Prevotella*, and the concentration of these metabolites was more pronounced in the group fed whole lingonberries. Strong positive correlations were detected between *Bifidobacterium* and these metabolites (r = 0.74, 0.74, 0.67 and 0.91 for CDCA, UDCA, α-MCA and HDCA, respectively) (Fig. 5). Furthermore, *Prevotella* showed positive correlation with UDCA and α-MCA (Supplementary Fig. 1).

On the other hand, DCA and T-DCA, which were most concentrated in the group fed the insoluble lingonberry fraction, made one cluster together with *S24-7*, *Mucispirillum* and *Ruminococcus* (Fig. 4). *S24-7* was negatively correlated with T-α-MCA and T-UDCA with r-values of −0.074 and −0.76, respectively (P < 0.001), while *Mucispirillum* was positively correlated with T-DCA (r = 0.78, P < 0.001) (Fig. 5). However, this bacterium showed negative correlation with T-α-MCA (Supplementary Fig. 1). Furthermore, DCA and TDCA were also positively correlated with *Ruminococcus*, while this bacterium showed negative correlation with T-UDCA (Supplementary Fig. 1).

To the left side of the PLS plot, T-α-MCA, ω-MCA and T-UDCA grouped together with *Bacteroides* and *Parabacteroides* (Fig. 4). A positive correlation was seen between *Bacteroides* and Tα-MCA, ω-MCA and T-UDCA (Supplementary Fig. 1). *Parabacteroides* only showed positive correlation with T-α-MCA and T-UDCA. These two microbiota strains showed negative correlation with DCA and T-DCA (Supplementary Fig. 1).

### Hepatic gene expression of Nr1h4, Cyp8b1 and Nr0b2

**Figure 6:**
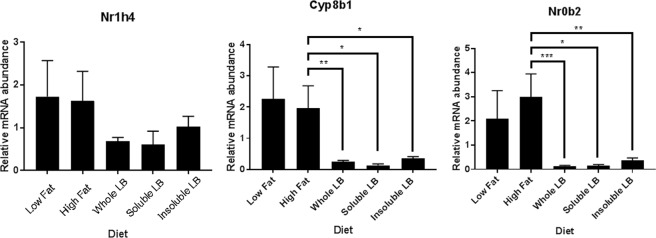
Hepatic gene expression of Nr1h4, Cyp8b1 and Nr0b2 in Apoe*−/−* mice fed low-fat (LF) or high-fat (HF) control diets, or HF diets supplemented with different lingonberry fractions. *P < 0.05, **P < 0.01, ***P < 0.001, ****P < 0.0001 (Mann-Whitney U test).

To investigate the underlying mechanism/pathway responsible for the altered BA profile after intake of lingonberry fractions, quantitative RT-PCR was performed for three genes involved in BA metabolism. Gene expression of Nr1h4, Cyp8b1 and Nr0b2 in the liver was not significantly different between LF and HF groups (Fig. 6). In contrast, all lingonberry groups exhibited significantly lower expression of Cyp8b1 and Nr0b2 compared to the HF control group and albeit not significant, the same trend was observed for Nr1h4 expression (Fig. 6).

## Discussion

The aim of the present study was to investigate the applicability of an UHPLC-MS method for determination of serum BAs in Apoe−/− mice fed diets containing different lingonberry materials, and whether the BA profile was related to gut microbiota composition. The UHPLC-MS method was successfully applied for quantification of the major and minor BAs present in the serum of mice. The method has been validated in terms of sensitivity, accuracy, precision, repeatability and reporting limit (RL) and gave particular information about BA composition and concentration in serum of mice fed diets containing different lingonberry samples.

It should be kept in mind that Apoe−/− mice have a BA profile different to wild type mice with, for example, higher concentrations of hepatic MCA and CDCA^47^. However, this is of less importance in the present study as the primary interest was not to determine the amount of BAs in absolute figures, but rather to rank and compare the effects of different diets. The results presented in this study apply only to the Apoe−/− mouse model, and the wild type mice may respond differently to lingonberries, although they respond with the same gut microbiota. The design of the study resulted in five groups including two control diets containing cellulose in LF and HF settings and three HF diets containing whole lingonberries or soluble and insoluble lingonberry fractions separated according to Jakobsdottir *et al*.^46^. It has been reported that about 95% of the BAs in the serum of mice are conjugated with taurine^5^, which is in agreement with the present study where we found very low values for glycine-conjugated BAs and, consequently, a lack of differences. However, the amount of taurine-conjugated BAs was affected considerably by the type of diet.

With regard to unconjugated BAs, the group fed the whole lingonberry diet had the best BA profile. Thus, the serum concentration of CDCA, a primary BA that has been reported to reduce plasma LDL levels and dissolve gallstones in humans^48,49^, was highest with this material. Furthermore, MCAs, only present in rodents, in which β-MCA has been reported to dissolve cholesterol gallstones in C57L/J mice^4^, were all generally higher in the whole lingonberry group compared to other groups. The amount of UDCA was also significantly higher in this group. This is interesting as UDCA has been reported to improve insulin resistance in a diabetic mice model^18^ and to have anti-inflammatory effects in an experimental rat model and human cancer cells^50,51^, due to its ability to decrease gut permeability. Notably, the concentration of DCA, known as a colorectal cancer promoter in humans^52,53^, was lower in the group fed with whole lingonberries. Interestingly, the group fed the whole lingonberries exhibited the highest amount of the conjugated BAs, T-UDCA and T-α-MCA. In this respect it is important to mention that T-UDCA has been shown to have neuroprotective effects in experimental mouse models of Alzheimer’s and Parkinson’s diseases^54,55^. The positive results in the whole lingonberry group may be due to the nutritional composition of these berries, consisting of specific antioxidants such as polyphenols but also dietary fiber, both known for their positive health effects^56,57^. A mixture of dietary fibre has previously been shown to be more beneficial for the colon formation of SCFAs by the microbiota, such as butyric acid, than isolated and pure dietary fibre^58^. It has been reported that about 69% of dietary fibre in lingonberry is insoluble, with approximately half of this amount being cellulose, while the soluble lingonberry fraction (approximately 31%) consists mainly of uronic acids and arabinose^44^. One explanation to the positive effect of dietary fibre can be by entrapping bile acids throughout the small intestine, reducing their reabsorption into the liver and, consequently affecting bile acid and cholesterol metabolism. Another explanation for the observed positive effect, could be that the fibre in lingonberries altered the gut microbiota composition and the formation of gut metabolites such as SCFAs. Antioxidant may also reach colon and being fermented by the microbiota.

Fewer effects were seen with the two lingonberry fractions. With the soluble lingonberry fraction, T-CA had most pronounced effects, and the concentration was higher in this group than in other groups. T-CA has been shown to have glucose-lowering effects in humans^59^ and immunoregulatory properties in normal Kunming mice^60^. The higher concentration of T-CA in this group is notable as the diet contained smaller amounts of dietary fiber from lingonberries. On the other hand, this fraction most probably contains benzoic acid as is completely soluble. In the group fed the insoluble lingonberry fraction, the concentration of UDCA, T-UDCA, T-α-MCA and ω-MCA was lower compared to the group fed the whole lingonberry diet. Thus, the increase of specific BAs seen in the whole lingonberry diet was not reflected with the lingonberry fractions. The reason for that is not known but could be due to that whole lingonberries contain a mixture of bioactive compounds that interplay with each other, modifying the microbiota composition, or that the fiber or other bioactive components have been modified/diluted in some way during the fractionation procedure, despite the fact that the fractions were quantitatively collected.

Berries, particularly lingonberries, have been reported to alter the gut microbiota, thus preventing inflammation^61^, reducing atherosclerosis^44^ and improving the hepatic function in mice^62^. Hence, we investigated further whether there were associations between the BA profiles induced by the different diets and gut microbial taxa. Bacterial results indicated that whole lingonberry provided a better bacterial profile compared with soluble and insoluble fractions. In this respect, *Bifidobacterium*, which is often used as probiotics^63,64^ and *Prevotella*, which has been shown to improve glucose metabolism in healthy subjects^65^, were most abundant in mice fed the whole lingonberry diet than in mice fed diets containing the lingonberry fractions. These bacteria were associated with CDCA, UDCA and α- MCA, often connected to beneficial health effects. The insoluble fraction of lingonberry showed a higher number of *Mucispirillum*, which has been reported to be higher in obese mice^66^ and associated with DCA and TDCA. Both these BAs correlated with *S24-7* and *Ruminococcus*, which are associated with inflammation and increased Alzheimer’s disease-like pathology in mice^67,68^. Additionally, *Bacteroides* and *Parabacteroides*, which are the most abundant anaerobic bacteria in the gut and considered to be healthy microbes^69^, were associated with T-UDCA, T-α-MCA and ω-MCA.

To determine whether BA signaling pathways in the liver were altered by lingonberry diets, hepatic gene expression levels of the Nr1h4 (FXR), Nr0b2 (Small heterodimers partner, SHP) and Cyp8b1 genes were measured. The Cyp8b1 gene is involved in the regulation of the proportions of CA and CDCA formed, while the Nr1h4 gene encodes FXR, a nuclear BA receptor that is a master regulator of the synthesis of endogenous BAs^70^, and SHP is an orphan nuclear receptor and a target for FXR that is upregulated by BAs in the liver^71^. All three genes were downregulated by the lingonberry diets, which supports our findings of an altered BA profile in the gut. In addition, an extended analysis of the gut microbiota composition^72^ revealed that there was a decreased alpha diversity of the gut microbiota in mice that were fed lingonberries. This may indicate that lingonberries stimulate certain microbial taxa, while inhibiting others, thus leading to an altered BA profile in the gut and subsequent decreased BA gene expression in the liver.

In conclusion, HF diet increased the serum concentration of individual BAs, which confirms the role of BAs in the body in terms of fat elimination. Among the different lingonberry diets, the whole lingonberries had the most considerable effects on the concentration of both unconjugated and conjugated BAs, by increasing the amount of CDCA, UDCA, α-MCA, ω-MCA, T-α-MCA and T-UDCA, and decreasing the concentration of DCA and T-DCA. In addition, the whole lingonberries induced the most beneficial BA composition in blood, which associated positively with the abundance of *Bifidobacterium* in the gut.

## Methods

### Chemicals and reference standards

BA standards including cholic acid (CA), chenodeoxycholic acid (CDCA), deoxycholic acid (DCA), lithocholic acid (LCA), ursodeoxycholic acid (UDCA) and hyodeoxycholic acid (HDCA) were purchased from Sigma-Aldrich Chemicals Co (Steinheim, Germany). α-muricholic acid (α-MCA), β-muricholic acid (β-MCA), ω-muricholic acid (ω-MCA), tauro-cholic acid (T-CA), tauro-chenodeoxycholic acid (T-CDCA), tauro-deoxycholic acid (T-DCA), tauro-lithocholic acid (T-LCA), tauro-ursodeoxycholic acid (T-UDCA), tauro-α-muricholic acid (T-α-MCA), tauro- β-muricholic acid (T-β-MCA), glyco-cholic acid (G-CA), glyco-chenodeoxycholic acid (G-CDCA), glyco-deoxycholic acid (G-DCA), glyco-lithocholic acid (G-LCA), glyco-ursodeoxycholic acid (G-UDCA) and deuterated internal standards (IS) lithocholic acid-2,2,4,4-D4 (LCA-D4), deoxycholic acid-2,2,4,4-D4 (DCA-D4) and glycochenodeoxycholic acid-2,2,4,4-D4 (GCDCA-D4) were obtained from Steraloids, Inc. (Newport, RI, USA). Methanol HPLC grade was from Merck (Darmstadt, Germany). Ultra-pure reagent water was purified by a Milli-Q gradient system (Millipore, Bedford, MA, USA). Acetonitrile LC-MS grade was from VWR and formic acid LC-MS grade was purchased from Sigma Aldrich Chemicals Co.

### Animals

Apoe−/− male mice, 7 weeks of age (Taconic Bioscience Inc. Silkeborg, Denmark), with an initial weight of 21.9 ± 0.2 g were randomly divided into 5 groups (n = 7) and assigned to one of the five diets. The mice were adapted to the environment (22 °C, 12 h light and 12 h dark cycle) and fed the chow diet for 7 d before starting the experimental period. The mice were then fed the actual diet for 8 weeks. However, the mice fed the soluble lingonberry fraction lost a substantial amount of body weight after just a couple of days. To recover, the mice returned to the chow diet for a couple of weeks and subsequently the mice were fed a diet in which the proportion of the soluble lingonberry fraction was reduced to 25% of the total fiber (i.e. 15 g/kg), by mixing the HF control diet (75%) with the soluble lingonberry fraction diet (25%). This part lasted the last two weeks of the experiment. After the experimental period, the animals were anesthetized using isoflurane, and serum samples were taken from the heart. Serum samples were kept at −80 °C until analysis. Cecum samples for gut microbiota characterization were collected with sterile instruments and frozen at −80 °C until analysis

### Approval of animal study

The Ethics Committee for Animal Studies (Review panel III) at Lund University approved the experiment (application number: M 114-15). All experiments were performed according to regulations issued by Lund University.

### Diets

Freeze-dried lingonberries (*Vaccinium vitis-idaea*) were obtained from Skogsmat in Uddeholm AB (Karlstad, Sweden). Some of the freeze-dried berries were separated based on their water solubility at pH 2 into insoluble and soluble fractions according to Jakobsdottir *et al*.^46^ and then freeze-dried again. The freeze-dried materials (whole lingonberries and isolated insoluble and soluble fractions of lingonberries) were ground by hand in a mortar and incorporated into three test diets with a high-fat content. Furthermore, two control diets containing cellulose in either a low-fat (LF, 50 g/kg, dry weight basis, dwb) or high-fat (HF 200 g/kg, dwb) settings were included in the experiment. The fiber level was 60 g/kg (dwb) in all diets. All diets were prepared by Research Diets Inc (New Brunswick USA) and contained the same amount of added protein (casein 154 g/kg, dwb), sucrose (51 g/kg, dwb), soy bean oil (50 g/kg), dwb), mineral mixture (35 g/kg, dwb), vitamin mixture (10 g/kg, dwb), cystine (1.8 g/kg, dwb) and t-butylhydroquinone (0.1 g/kg, dwb). Corn starch was used to adjust dry matter content. A detailed description of the diets can be found in a study by Marungruang N *et al*.^72^.

### Preparation of standard solutions, calibration curves and serum samples

A standard stock solution of each free and conjugated BA was prepared in a concentration of 1 mg/mL in methanol. To be able to make solutions for calibration points, mixture solutions of all free BAs and conjugated BAs were prepared separately, at a concentration of 50 µg/mL in methanol. A mixed solution of three deuterated IS containing a concentration of 400 ng/mL LCA-D4, 1200 ng/mL DCA-D4 and 4000 ng/mL GCDCA-D4 was also made and used for calibration points. This mixed IS solution was diluted seven times and used for spiking the serum samples. Standard solutions for a six-point calibration curve ranging from 5 to 10000 ng/mL were prepared in methanol by serial dilutions of the BA mixture solutions. The deuterated IS mixed solution was kept cold, to be used for protein precipitation. All solutions were stored at −18 °C until used.

Serum samples, which were kept at −80 °C, were allowed to thaw at room temperature, then prepared by precipitation of protein by methanol and analyzed by UHPLC-MS. Serum samples, 50 µL, were spiked with 175 µL of the diluted IS mix solution. The samples were vortexed for 30 s, and then incubated at −18 °C for 20 min. After centrifugation at 13,000 g for 15 min, the supernatant was collected and transferred to clean tubes and dried at 60 °C in a Mivac concentrator (Kovalent, Sweden) for 45 min. The residue was then reconstituted in 100 µL of Millipore water and transferred into vials to be injected onto the UHPLC-MS system.

### Apparatus and instrumental conditions

Serum samples were analyzed using a Waters Acquity UHPLC system (Waters Corporation, Milford, MA). The samples were injected onto a Waters Acquity UHPLC CSH-C18 column (1.7 µm, 2.1 × 100 mm; Waters Corporation) equipped with an Acquity UHPLC CSH-C18 VanGuard pre-column (1.7 µm, 2.1 × 5 mm; Waters Corporation). The temperature of the column was 65 °C and the flow rate was 0.55 mL/min. The mobile phases consisted of 0.1% formic acid in water (eluent A) and 0.1% formic acid in acetonitrile (eluent B). The 16-min gradient elution was performed as follows: 32–39% B (0–4 min), 39–40% B (4–4.1 min), 40–45% B (4.1–8 min), 45–60% B (8.0–8.5 min), 60–80% B (8.5–12.5 min). At 12.5 min, the amount of B was kept constant at 80% for 0.1 min and followed by a quick increase to 95% for 0.1 min. Thus, at 12.7 min, the concentration of B was 95% and in 0.1 min it had dropped to 32% and maintained this concentration for 3 min for equilibration and column conditioning. The sample injection volume was 3 µL and the autosampler temperature was set to 4 °C.

The MS analysis was done using a Waters Xevo G2 QTOF (Waters MS Technologies, Manchester, UK) equipped with an ESI source and operated in the negative ion mode. A capillary voltage of 2.5 kV, sample cone voltage of 70 V, a source temperature of 120 °C and a desolvation temperature of 450 °C were applied. Desolvation and cone gas flows were set as 800 and 50 L/h (N2), respectively. Data were collected in centroid sensitivity mode in the range 100–800 *m/z*, with lockspray scan collected every 30 s and an average of 3 scans to perform mass correction. For quantification, the m/z of each molecular ion was used with a tolerance of 0.03 Da.

### Method validation

To validate the analytical method, a higher volume of serum sample was needed. Thus, serum samples from rats (from a previous study) were pooled and used for testing the method. Serum sample from rats was the best choice for validating the extraction and chromatographic methods as they contain all forms of BAs (free, glycine and taurine-conjugated BAs). 50 µL of rat serum samples were spiked at low µg/mL levels. All analyses were done in triplicate.

The linearity of the calibration curve was evaluated based on the following calibration standard concentrations: 5 ng/mL, 10 ng/mL, 50 ng/mL, 100 ng/mL, 1000 ng/mL, 5000 ng/mL and 10000 ng/mL. Each level of concentration was analyzed in triplicate. The method was linear with coefficient of determination (R^2^) in the range of 0.9931–0.9999 (Table 2).

**Table 2:** Chemical characteristics of BAs and IS, retention time and R^2^ with the applied method.

Bile acids	Formula	MW	[M –H]^−^	IS	Rt (min)	R^2^
**Unconjugated**						
CA	C_24_H_40_O_5_	408.3	407.28	GCDCA-D4	4.86	0.9980
α-MCA	C_24_H_40_O_5_	408.3	407.28	GCDCA-D4	3.13	0.9931
β-MCA	C_24_H_40_O_5_	408.3	407.28	GCDCA-D4	3.46	0.9977
ω-MCA	C_24_H_40_O_5_	408.3	407.28	GCDCA-D4	2.88	0.9978
CDCA	C_24_H_40_O_4_	392.3	391.28	DCA-D4	8.50	0.9997
DCA	C_24_H_40_O_4_	392.3	391.28	DCA-D4	8.85	0.9999
HDCA	C_24_H_40_O_4_	392.3	391.28	GCDCA-D4	5.50	0.9995
UDCA	C_24_H_40_O_4_	392.3	391.28	GCDCA-D4	5.21	0.9994
LCA	C_24_H_40_O_3_	376.3	375.29	LCA-D4	10.59	0.9955
**Glycine-conjugated**						
G-CA	C_26_H_43_NO_6_	465.7	464.3	GCDCA-D4	3.03	0.9987
G-LCA	C_26_H_43_NO_4_	433.4	432.31	DCA-D4	9.18	0.9986
G-CDCA	C_26_H_43_NO_5_	449.6	448.31	GCDCA-D4	5.40	0.9955
G-DCA	C_26_H_43_NO_5_	449.6	448.31	GCDCA-D4	5.92	0.9962
G-UDCA	C_26_H_43_NO_5_	449.6	448.31	GCDCA-D4	2.99	0.9987
**Taurine-conjugated**						
T-CA	C_26_H_45_NO_7_S	515.3	514.28	GCDCA-D4	5.06	0.9960
T-α-MCA	C_26_H_45_NO_7_S	515.3	514.28	GCDCA-D4	2.93	0.9977
T-β-MCA	C_26_H_45_NO_7_S	515.3	514.28	GCDCA-D4	3.09	0.9966
T-UDCA	C_26_H_45_NO_6_S	499.3	498.29	GCDCA-D4	4.92	0.9959
T-CDCA	C_26_H_45_NO_6_S	499.3	498.29	DCA-D4	8.05	0.9992
T-DCA	C_26_H_45_NO_6_S	499.3	498.29	DCA-D4	8.85	0.9995
T-LCA	C_26_H_45_NO_5_S	483.3	482.29	LCA-D4	11.16	0.9936

Serum samples of rats were used for method validation.

In order to measure precision, 20 µL serum samples were spiked with IS, and intra-day and inter-day precisions were determined based on the peak area of each BA. According to Table 3, the intra-assay coefficient of variation (CV (%)) ranged from 1.7% (G-LCA) to 10.2% (T-LCA) and the inter-assay, calculated on two different days with a total of six runs, yielded a CV of 2.6% (CA) to 39.5% (T-CDCA).

**Table 3:** Precision, recovery and reporting limit and signal-to-noise ratio (S/N) for each BA.

Bile acids	Intra-assayCVs* (%)	Inter-assayCVs** (%)	Recovery (%)1000 ng/mL	Reporting limit(RL) ng/mL	S/N instandard
**Unconjugated**					
CA	3.2	2.6	142	5	84
α-MCA	2.8	23.6	101	5	48
β-MCA	2.9	17.3	114	5	85
ω-MCA	2.2	18.8	128	5	81
CDCA	3.4	38.3	103	5	14
DCA	4	19.3	115	5	54
HDCA	2.9	9.7	152	5	20
UDCA	3.5	27.3	95	5	24
LCA	8.9	10.6	117	50	16
**Glycine- conjugated**					
G-CA	3.4	4	137	5	47
G-LCA	1.7	31	78	5	22
G-CDCA	3.7	17.2	116	5	60
G-DCA	4.1	25	108	5	14
G-UDCA	2.7	27.6	103	5	19
**Taurine-conjugated**					
T-CA	3.6	14.7	120	5	16
T-α-MCA	4.1	27.5	111	5	61
T-β-MCA	4.1	17.2	106	5	51
T-UDCA	3.7	20.2	108	5	34
T-CDCA	3.9	39.5	90	5	37
T-DCA	4.8	38.7	86	5	50
T-LCA	10.2	22.7	124	10	14

Serum samples of rats were used for method validation. *(n = 3) **(n = 6).

The accuracy was defined in terms of recovery. The recoveries of all unconjugated and conjugated BAs were assessed by spiking them to 50 µL serum samples at the concentration of 1000 ng/mL and were in the range 78 to 152% (Table 3). The average recoveries for the unconjugated, glycine and taurine-conjugated BAs were 118, 108 and 106%, respectively, which were higher than recovery values (80, 98 and 86%) reported by Want *et al*. using a similar methodology^26^.

Reporting limit (RL) was calculated using the lowest calibration point. All BAs had RL of 5 ng/mL, except for LCA and T-LCA, which showed RL of 50 ng/mL and 10 ng/mL, respectively. Furthermore, the signal-to-noise ratio of all BAs in the standard solution was between 14 and 85 (Table 3).

### Analysis of gut microbiota composition

Cecal samples from the groups fed whole lingonberries (n = 7), soluble (n = 3) and insoluble lingonberry fractions (n = 7) were analyzed using 16 S rRNA gene sequencing as described previously^24^.

### Real-time quantitative Reverse Transcription-PCR (RT-qPCR)

For extraction of total RNA, mice livers were lysed in RLT buffer and disrupted and homogenized using a TissueLyser II and 5 mm diameter steel beads (Qiagen, Valencia, CA, USA). RNA purification was performed with the Qiagen RNeasy mini kit including an on column DNase treatment to remove genomic DNA (Qiagen, Valencia, CA, USA), as recommended by the manufacturer. Total RNA was assessed for its integrity and purity using agarose gel electrophoresis and Nanodrop spectrophotometer (Nanodrop Technologies, Wilmington, DE, USA), respectively. For each liver sample, cDNA was generated from 1 µg total RNA using the RevertAid First Strand cDNA synthesis kit (ThermoScientific, Waltham, Massachusetts, USA) with random hexamer primers. The product of the first strand cDNA was amplified by qPCR. Housekeeping gene primers were designed using primer 3 software and genes of interest using the universal probe library (Roche, Basel, Switzerland) (Table 4). Total RNA equivalents of cDNA (20 ng) were used in each qPCR amplification, run in duplicate on the same plate. Detection of the PCR product was carried out by the CFX384 Real-Time PCR system (Biorad, Hercules, CA, USA) using the DNA-binding dye SYBR Green I. To account for possible variation related to DNA input amounts or the presence of PCR inhibitors, two housekeeping genes (Hprt and Ubc) were selected (by GeNorm) out of 6 tested candidate reference genes (Table 4).

**Table 4:** Primer sequences used in qPCR studies.

Gene	Forward primer	Reverse primer	Amplicon length
**Reference genes**			
Actb	GCTTCTAGGCGGACTGTTACTGA	GCCATGCCAATGTTGTCTCTTAT	101 bp
Actg	ACCAACAGCAGACTTCCAGGAT	AGACTGGCAAGAAGGAGTGGTAA	76 bp
Hmbs	GAAACTCTGCTTCGCTGCATT	TGCCCATCTTTCATCACTGTATG	101 bp
Hprt	CCTAAGATGAGCGCAAGTTGAA	CCACAGGACTAGAACACCTGCTAA	86 bp
Rpl13a	CCTGCTGCTCTCAAGGTTGTT	TGGTTGTCACTGCCTGGTACTT	103 bp
Ubc	AGGTCAAACAGGAAGACAGACGTA	TCACACCCAAGAACAAGCACA	101 bp
**Target genes**			
cyp8b1	CAGGAAGTTCCGTCGATTTG	GGCCCCAGTAGGGAGTAGAC	60 bp
Nr0b2	AAAGGACCAACCAATCTCCA	GGGAGTTAGTCTTTCCCATGAGT	70 bp
Nr1h4	GAAAATCCAATTCAGATTAGTCTTCAC	CCGCGTGTTCTGTTAGCAT	106 bp

### Data analysis

All analyses of standard solutions for method validation were performed in triplicate, while the analyses on mice serum samples were done in duplicate due to low amounts of serum sample obtained from each mouse. The results are presented as means and their standard error means (SEM).

Minitab statistical software (release 17, MINITAB Inc, State College, PA, USA) and GraphPad (Prism 7) were used for data evaluation. To determine differences in mean values between groups, one-way ANOVA was used. Dunnett’s procedure was then applied to evaluate significances between groups. Values from the LF control group were used as reference (the basic value), and those from the HF control as the worst value. Thus, all values were compared to the LF control diet and the significances were marked with a cross (†) (Table 1). Furthermore, to see whether the lingonberries had an effect on BA concentrations, these diets were also compared to the HF control diet and significances were marked with an asterisk (*) (Table 1). Finally, to compare significant differences between the lingonberry diets, one-way ANOVA followed by Tukey’s test was used and the significances were marked with an asterisk (*) (Fig. 3). MassLynx 4.1 (Waters) was used for data acquisition and quantification.

Correlation between BAs and gut bacterial genera in groups fed different lingonberry fractions were analyzed with SIMCA-14.0 software (Umetrics, Umeå, Sweden) and illustrated in a PLS plot (Fig. 4). A score scatter plot was used to show the position of each diet.

P-values of gene expression results were calculated by means of a Mann-Whitney U test, using GraphPad (Prism 7).

## Supplementary information

Supplementary Fig. 1

## Data Availability

All data generated or analyzed during this study are included in this published article.
