# Supplementary material for: Determination of free and conjugated bile acids in serum of Apoe(−/−) mice fed different lingonberry fractions by UHPLC-MS
Source: Sci Rep. 2019 Mar 7;9:3800. doi: 10.1038/s41598-019-40272-8 (PMC6405994; doi:10.1038/s41598-019-40272-8)
Supplement: Supplementary file 1 — Supplementary Fig. 1 [file 41598_2019_40272_MOESM1_ESM.pdf]

Determination of free and conjugated bile acids in serum of Apoe (-/-) mice fed different lingonberry fractions by UHPLC-MS

Tannaz Ghaffarzadegan[a,c], Sofia Essén[b], Phebe Verbugghe[c], Nittaya Marungruang[a,c], Frida Fåk Hållenius[a,c], Margareta Nyman[a,c] and Margareta Sandahl[b]

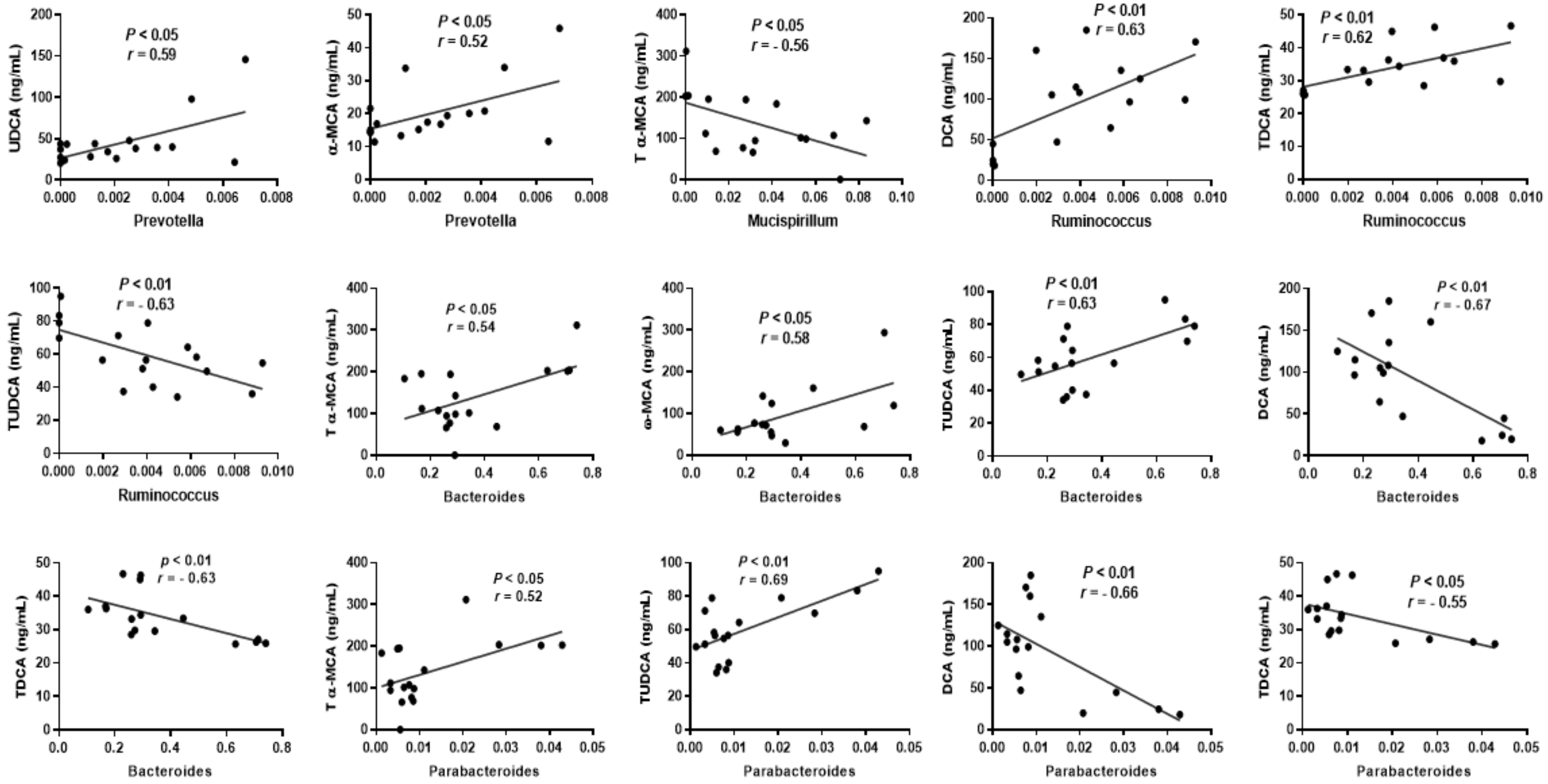

Supplementary Fig 1. Moderate correlation between BAs and selected microbiota in Apoe -/- mice (r-values > 0.5). X-axis represents the relative abundance of each microbiota.
